# Shared but Threatened: The Heritage of Wild Food Plant Gathering among Different Linguistic and Religious Groups in the Ishkoman and Yasin Valleys, North Pakistan

**DOI:** 10.3390/foods9050601

**Published:** 2020-05-08

**Authors:** Muhammad Abdul Aziz, Arshad Mehmood Abbasi, Zahid Ullah, Andrea Pieroni

**Affiliations:** 1 University of Gastronomic Science, Piazza Vittorio Emanuele II 9, 12042 Pollenzo, Bra, Italy; m.aziz@studenti.unisg.it; 2 Department of Environmental Sciences, COMSATS University Islamabad, Abbottabad Campus, 22060 Abbattabad, Pakistan; arshad799@yahoo.com; 3 Center for Plant Science and Biodiversity, University of Swat, Kanju 19201, Pakistan; zahidtaxon@uswat.edu.pk

**Keywords:** ethnobotany, ethnobiology, local ecological knowledge, local food knowledge, Gilgit-Baltistan

## Abstract

A wild food ethnobotanical field study was conducted in the Ishkoman and Yasin valleys, located in the Hindukush Mountain Range of Gilgit-Baltistan, northern Pakistan. These valleys are inhabited by diverse, often marginalized, linguistic and religious groups. The field survey was conducted via one hundred and eighty semistructured interviews to record data in nine villages. Forty gathered wild food botanical and mycological taxa were recorded and identified. Comparative analysis among the different linguistic and religious groups revealed that the gathered wild food plants were homogenously used. This may be attributed to the sociocultural context of the study area, where most of the population professes the Ismaili Shia Islamic faith, and to the historical stratifications of different populations along the centuries, which may have determined complex adaptation processes and exchange of possibly distinct pre-existing food customs. A few wild plants had very rarely or never been previously reported as food resources in Pakistan, including *Artemisia annua*, *Hedysarum falconeri, Iris hookeriana*, *Lepidium didymium* and *Saussurea lappa.* Additionally, the recorded local knowledge is under threat and we analyzed possible factors that have caused this change. The recorded biocultural heritage could, however, represent a crucial driver, if properly revitalized, for assuring the food security of the local communities and also for further developing ecotourism and associated sustainable gastronomic initiatives in the area.

## 1. Introduction

Food systems and their elements and relationships are subject to constant modifications by wider processes of change linked to regional and global trends. Moreover, with the natural environment being the basis of food production, food systems should always be understood as coupled social-ecological systems [1]. Mountain communities are considered particularly vulnerable to food insecurity, and their vulnerability is sometimes assumed to be increasing because of difficult conditions for agricultural production, social and political marginalization, and the negative impacts of climate change. A number of ethnographic studies have pointed out that food systems in mountains are fragile, dynamic and multifaceted, relying on diverse farm and off-farm sources of livelihood while being subject to manifold social, economic, political and ecological changes [2,3,4,5].

Wild food plants (WFPs) have provided a key source of food to humans since prehistoric times, although their importance in the human diet has diminished, first with agricultural expansion and later more dramatically with industrialization and urbanization processes [6]. While traditional/local ecological knowledge (LEK) identifies the complex body of understanding/knowledge, practices and beliefs (UKPB) that human societies have developed in inextricable relationships with their natural environment, and which is dynamic and coevolving with social and ecological changes [7], we believe that traditional/local food knowledge (LFK) refers to the UKPBs related to the environmental foodscape (agroecosystems where ingredients are produced), as well as the culinary practices/skills, the local recipes, and the social contexts of food consumption within a given community.

Both LEK and LFK, therefore, represent an integral part of sustainable and sovereign local food systems that need to be dynamically preserved; however, for a few decades, global environmental and socioeconomic changes, and especially food industrialization, commodification and globalization have had a negative impact on LEK and LFK, which are often considered vanishing and somehow eroded, since they are often perceived as part of a fading, orally-transmitted, local biocultural heritage. Nevertheless, since environmental and social relationships are constantly changing, LEK and LFK are actually not static but should be more correctly seen as a mutating complex that are continuously renegotiated [8,9].

Over the last decade, ethnobiologists have documented LEK on gathering and consuming WFPs in very different regions of the world ([10,11,12,13,14], and references therein), in order to provide concrete tools for fostering sustainable trajectories of rural development, or even sometimes for contributing to food security [15,16,17,18,19,20,21,22,23]. More recently, especially in Europe and the Middle East, an interesting trajectory of ethnobotanical research has concerned the cross-cultural comparison of wild food plants used among various ethnic and religious groups or among diasporas [24,25,26,27,28,29,30]. This emerging area may provide reflections concerning the ways though which cultural factors influence the transmission, evolution and change of plant ingredient use in traditional cuisines.

Pakistan boasts various kinds of natural resources, but the country still experiences food shortages. According to a report of the global hunger index (GHI), the country is facing serious food security issues [31]. In recent years, various biophysical and socioeconomic factors have led to a depletion of natural resources across the Hindukush Himalayan region. This has resulted in a significant loss of ecosystem services, particularly in terms of soil nutrients, water and biomass, and the resultant decline in food productivity [32]. Gilgit-Baltistan (Northern Pakistan) represent a multicultural and multilingual reservoir, which is inhabited by various marginalized linguistic minorities [33] that have a close attachment to natural resources for their livelihood, largely based on small-scale horticultural and pastoralist activities. It is relevant to mention that in northern areas of Pakistan, the completion of the Karakoram Highway in 1979 has had an impact on the local communities [34,35,36] and there has been considerable economical change, which has led to the diversification of livelihoods. However, at the same time, there are several communities residing at higher elevations that still today can be reached only by foot, and have little reliance on markets to supply food. Therefore WFPs play a crucial role in these peripheral communities [37,38] and surrounding regions as well [39,40]. In recent decades, the rapid processes of “modernization” and changing lifestyle of mountain communities have led many locals to embrace the western-style way of food procurement via large-scale markets, and this phenomenon has been and still is detrimental to LEK and LFK on WFPs [26]. Moreover, the local perception of the effects of climate change already seems to be considerable in Gilgit-Baltistan [41], while its specific effect on LEK is still understudied.

The overarching aim of this research was therefore to document these threatened local knowledge systems related to WFPs, and to provide stakeholders a possibly useful baseline of data for revitalizing them. We specifically investigated the impact that linguistic and religious affiliations have on the gathering and consumption of WFPs in two remote valleys of Northern Pakistan. The objectives of the study were therefore: (a) to record traditional wild food plant uses among different linguistic and religious communities living in the Ishkoman Valley and the Yasin Valley; (b) to compare the same data with the pre-existing food ethnobotanical surveys conducted in Northern Pakistan and (c) to better understand the diachronic dynamics of change of LEK and LFK linked to WFPs in order to possibly promote this heritage in sustainable rural development programs.

## 2. Materials and Methods

### 2.1. Study Area and Communities

**Figure 1:**
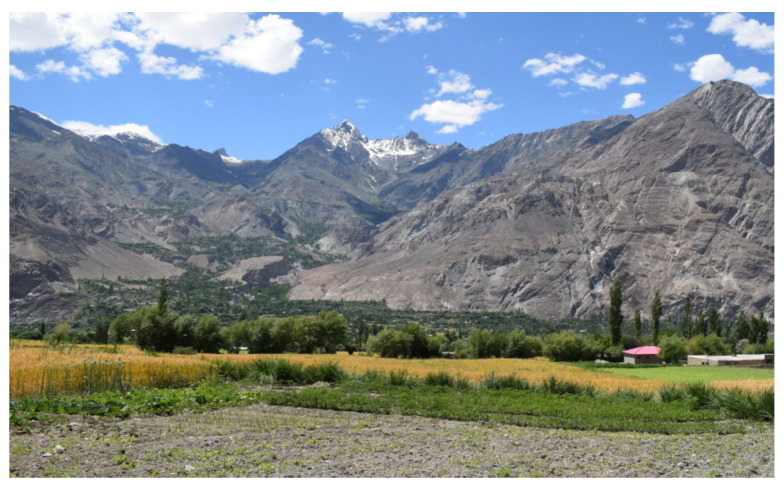
Landscape of the study area (an east-west view) with a wheat crop field. at Chatorkand, Ishkoman Valley, July 2019.

The study area is situated in the mountainous territory that is part of the Western Ghizar (or Ghizer) District in Gilgit-Baltistan, Northern Pakistan. The Ghizar District represents the westernmost part of the Gilgit-Baltistan region of Pakistan and is a crossroads between Gilgit and Chitral via the Shandur Pass, and also to China and Tajikistan via the Broghil Pass through the Ishkomen Valley. Ghizer District belongs to the Western Himalayan floristic region [42] (Figure 1) and has a humid continental climate (subtype Dwb) according to the Köppen classification [43]. The district is the home of four major linguistic groups: Shina, Kho, Burusho and Wakhi [44].

#### 2.1.1. Ishkoman Valley

The Ishkoman Valley is located in the transition zone between the Hindukush and Karakoram mountain ranges. In a historical perspective, Ishkoman figured as a regional political entity between the principalities of Hunza in the west and Yasin in the east. The northern boundary is contiguous with the Afghan controlled part of Wakhan. During the 20th century, the average growth rate of the population in Ishkoman steadily increased at a rate of 3% per year. Shina-speaking residents claim to be descendants of immigrants from Darel and Yasin, while additional migrants arrived from neighboring regions introducing other languages. In the central part of the valley, Khowar-speaking families from Ghizer, Turkho and Laspur (Chitral) took residence. In 1883, the ruler of Wakhan, Mir Ali Mardan Shah, fled his principality and took refuge under the protection of the Mehtar of Chitral. Mehtar Aman-ul-Mulk allocated barren tracts of land in the Karambar side valley to a growing group of Wakhi refugees [45]. In 1906, the total population of Ishkoman consisted of 1220 people, of which 390 claimed to be Khowar (32%), 377 Shina speakers (31%) and 453 Wakhi (37%) [46]. Wakhi habitations were clustered in Karambar. Shina speakers dominated the oldest settled parts of the upper Ishkoman River in addition to both banks of the lower valley where the Ishkoman borders Punial, a Shina speaking area. The Kho occupied the central fertile lands of Pakora, Chatorkhand and Dain. The three original settlement centers of importance continue to be the domain of Wakhi, Shina and Khowar speakers, while younger migrant groups have altered this pattern [44].

#### 2.1.2. Yasin Valley

Yasin valley is one of two valleys located in the middle of the western-central part of the mountainous belt of Northern Pakistan. Linguistic field research has found that the valley has remained the home of the speakers of the isolated Burushaski language [33,47,48,49]. Jettmar [50,51] asserted that there is evidence proving that the Burusho people descend from an archaic stratum of migrants or even the original inhabitants and that in later times Shina superseded and replaced Burusho in the Hunza and Yasin valleys. Researchers have claimed that it is highly likely that the arrival of Indic languages to the area started with the ancestors of present-day Kho and Shina speakers about a millennium ago and resulted in the occupation of the lower parts of the valleys; Gilgit and Chitral became their political centers from which further settlements spread into adjacent valleys. Previous research studies have shown that intra-montane migration was undertaken in order to search for cultivable land and grazing pastures. Significant migration within the mountain belt has taken place during the present century. New settlements were established in previously unoccupied territory either on barren terraces through irrigation or by converting temporary pasture settlements into permanent villages [44]. The literature indicates that for long periods Ghizer was under Chitrali rule, resulting in the migration of Kho people into the area from Chitral [44].

### 2.2. Field Study

**Figure 2:**
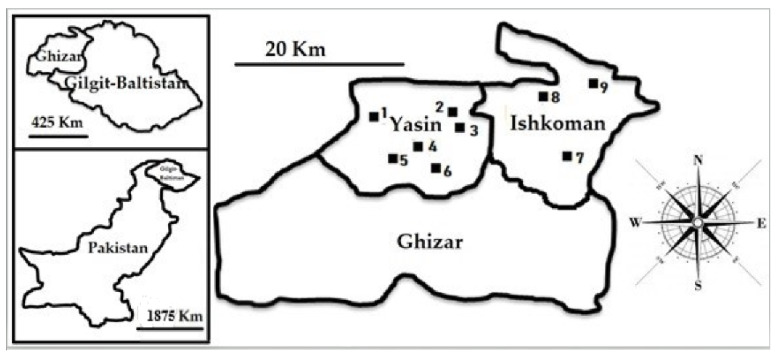
Map of the study area and visited villages: 1. Barkolti; 2. Sandi; 3. Ghojalti; 4. Sultan Abad; 5. Thawoos; 6. Yasin Khas; 7. Chatorkand; 8. Ishkoman Khas and 9. Imit.

A field ethnobotanical study was carried out from June to July 2019 in 9 mountain villages (Figure 2) in the Yasin and Ishkoman valleys of Gilgit-Baltistan, North Pakistan.

Information was gathered from different linguistic and religious groups that were dispersed in different villages across the valleys. Study participants, which were recruited through the snowball technique, were selected among middle-aged and elderly inhabitants (range: 52–69 years old), including farmers, shepherds and housewives who were considered possible knowledge holders. From each of the studied groups, twenty participants were selected for interviews, including both male and female community members.

Table 1 provides a brief summary of the characteristics of the visited villages and the considered sample.

**Figure 3:**
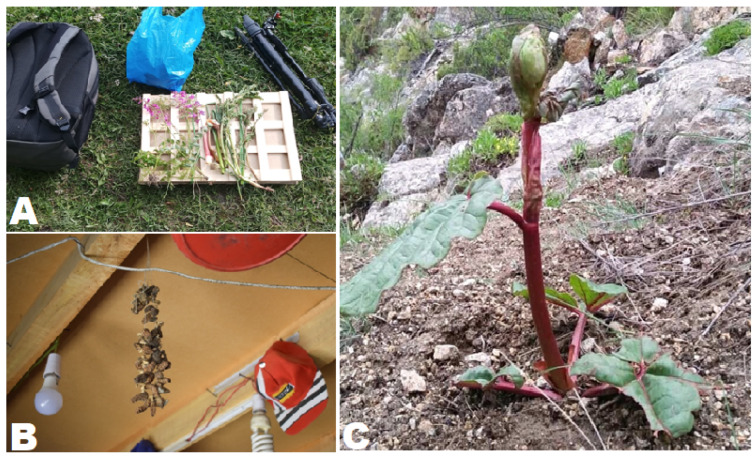
(**A**): A few flowering specimens collected for the herbarium; (**B**): *Morchella esculenta* hanging by a string at a local shop in Imit village and (**C**): *Rheum* sp.

Prior to each interview, verbal consent was obtained from the participants and the Code of Ethics adopted by the International Society of Ethnobiology [52] was followed. Semistructured interviews were conducted both in the Urdu language, as well as in the local language (with the help of a local translator). The interviews were focused on gathered and consumed wild food plants used as cooked vegetables, raw in salads, as snacks, as seasoning or for recreational teas. Specific questions were also asked concerning wild plants possibly used in dairy products or in lactic fermented foods, as well as the consumption of edible mushrooms. Moreover, we recorded information on some cultivated species, which locals considered “wild” or whose culinary use was unusual. For each of the free listed plants recorded during the study, the local name, gathering period and local food uses were documented. Additionally, qualitative ethnographic information was gathered via open-ended questions and participant observation. The quoted wild food taxa were then collected from the study area (Figure 3).

Collected plant taxa were identified by the third author using the Flora of Pakistan [53,54,55,56], and voucher specimens were deposited at the Herbarium of the Department of Botany, University of Swat, Khyber Pakhtunkhwa, Pakistan. Identification of the few wild plants for which it was not possible to collect vouchers was made on the basis of the folk name and detailed plant description only. Nomenclature followed The Plant List database [57] for each plant taxon and the Index Fungorum [58] for the mushroom taxon, while plant family assignments were consistent with the Angiosperm Phylogeny Website [59].

### 2.3. Data Analysis

Ethnobotanical taxa and their uses were compared among different community groups through proportional Venn diagrams, which were drafted using free software (http://bioinformatics.psb.ugent.be/webtools/Venn/). Data analysis was also carried out by calculating the Jaccard index (for each pair of considered communities), used for gauging the similarity and diversity of sample sets, following the application that González-Tejero et al. [60] designed for the use of this ecological index in the ethnobotanical domain. Moreover, for national data comparison, a detailed literature survey on the ethnobotany of wild food plants of Pakistan and Pamir was also conducted [26,37,38,39,40,61,62,63,64,65,66,67,68,69,70,71,72,73,74,75].

## 3. Results

### 3.1. Food System of the Studied Communities

**Figure 4:**
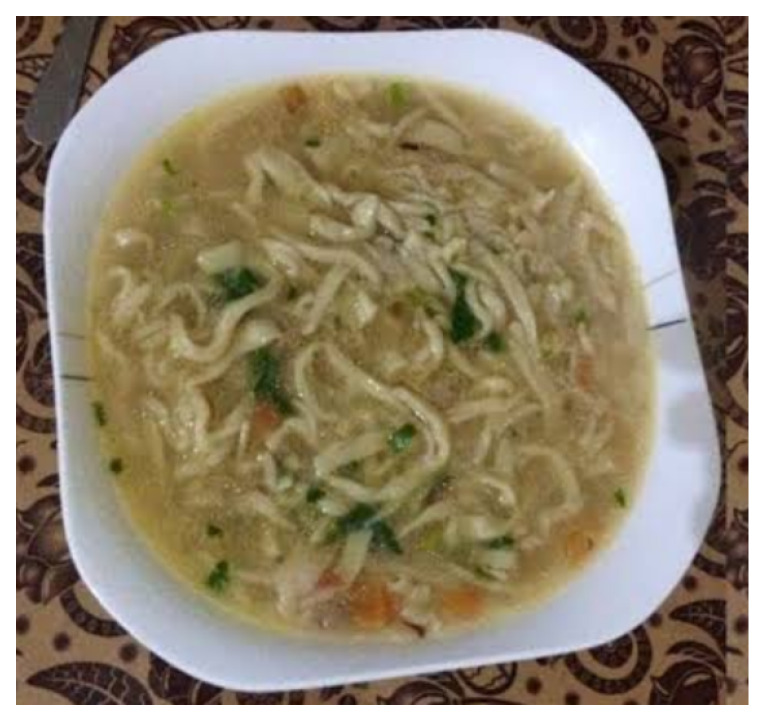
Traditional noodle soup known as Dawdoo, prepared in winter (photo courtesy of Asad Rahman).

The traditional food system of the studied communities was based on ingredients obtained from seasonal crops, as well as dairy products and the meat of sheep, cow and goat, as these communities have historically been attached to small-scale horticulture and livestock rearing. Almost every household in the study area had a small piece of land where the family cultivated different crops and vegetables. Cultivated crops contributed to the management of the traditional food system, which primarily consisted of consuming corn, wheat, buckwheat, pearl millet, barley and potatoes. Some important vegetables grown by locals in their home gardens or fields included cabbage, cucumbers, tomatoes, turnips, carrots, radishes, amaranth and lettuce. In the past, primary orchard foodstuffs, such as apricots and mulberry, were also supplemented with grains like barley, foxtail millet, buckwheat, fava bean and amaranth. Apart from these products, the most culturally salient customs of the people living in the area, and for which they are famous, are the frequent culinary uses of potato and rye. Different homemade food products were preserved and used at different times throughout the year. Some important dishes recorded in the study area include: Dawdoo, which is a famous noodle soup used especially in winter (Figure 4), Makoti (wheat flour combined with ground nuts and almonds and then cooked together), Terbat (wheat flour mixed with walnuts, almonds, and vegetable oil), Gyal (prepared by mixing wheat flour with butter and eggs), Chahn (wheat grain cooked with meat), Bappa (cooked wheat flour), Paqo (cooked wheat flour), Brat tiki (made by mixing wheat flour with butter and eggs), Chalpak (made by mixing local herbs with dough and oil), Mul (wheat flour combined with butter and then cooked), Molida (wheat flour mixed with milk and butter), Bayo-Cha or Trup Cha (salty tea mixed with milk) and Sharbat (wheat flour mixed with butter and then cooked).

All the above mentioned traditional dishes contained ingredients or products obtained from local small scale family-run horticultural and pastoralist activities, while the consumption of wild plants was considerable for those families living in the most isolated locations of the valleys.

### 3.2. Wild Food Plant Uses

The survey recorded forty plant and fungal taxa, which were used in the traditional food system across the two valleys (Table 2).

Among the thirty-nine reported botanical taxa, thirty-eight were taxonomically identified. The recorded taxa also included one fungal taxon. In addition, the plant taxa included a few cultivated food plants that were considered by locals to be “wild”, and/or they were used in unusual ways (these taxa are also included in Table 2). In the same table we also indicated the most quoted taxa for each considered religious and ethnic group. Among the recorded wild food plants, twenty-one taxa were cooked and consumed as vegetables. Some of the most frequently used plant species cooked as vegetables were *Allium carolinianum*, *Allium fedtschenkoanum*, *Amaranthus cruentus*, *Artemisia annua*, *Capparis spinosa*, *Eremurus himalaicus*, *Iris hookeriana*, *Lepidium didymium*, *Lepyrodiclis holosteoides*, *Taraxacum campylodes* and *Urtica dioica*.

Furthermore, thirteen taxa were consumed raw as snacks and a few plants were used in recreational herbal teas and as seasonings. Wild plant taxa used in herbal drinks or seasonings that were frequently mentioned included the seeds of *Carum carvi,* the bark of *Elaeagnus angustifolia* and the aerial parts of *Thymus linearis*. Wild food plants from the study area, which were rarely quoted included: *Medicago sativa, Ribes* spp., *Rubus fruticosus, Rumex dentatus*, *Saussurea lappa*, *Silene conoidea*, *Silene vulgaris*, *Sorbus* sp. and *Tulipa* sp. It is worth mentioning that only two plants, namely *Allium carolinianum* and *Allium fedtschenkoanum*, were frequently quoted by all the investigated groups.

**Figure 5:**
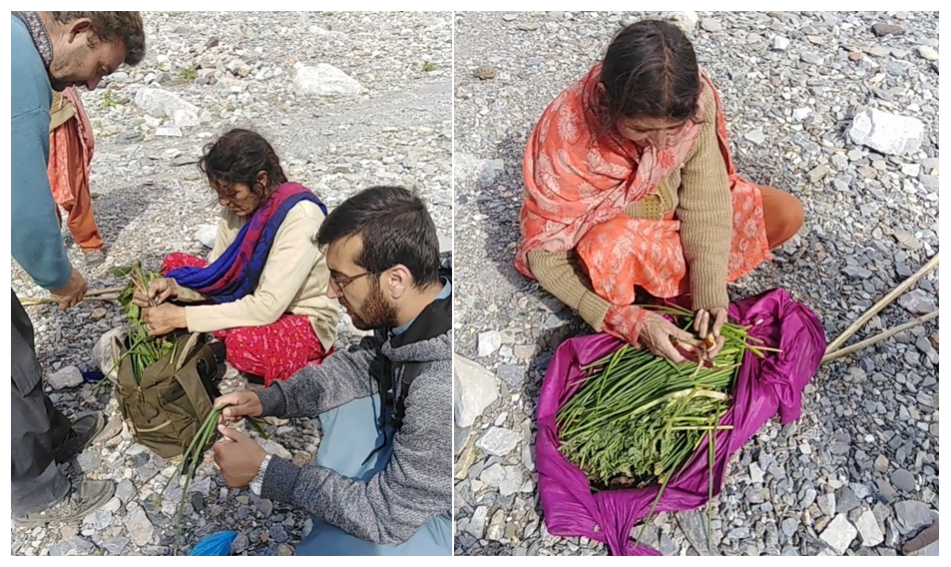
Locals and the first author after having gathered wild food plants in *Nalla*.

During the course of the ethnobotanical survey, local communities did not mention any wild plants used in fermentation. Study participants stated that there are certain plants that were mainly collected during the late spring, including *Allium carolinianum, Allium fedtschenkoanum, Bergenia stracheyi, Capparis spinosa, Carum carvi, Eremurus himalaicus*, *Rheum* spp. and *Thymus linearis*. Some of the study participants also mentioned that *Capparis spinosa*, which could usually be found near houses in villages where it was mostly gathered by women and children, was also sold in the market as it was an important medicinal plant. Study participants described certain plant species, which were collected in the mountains when needed for mainly medicinal purposes. An important spot for the collection of wild plants is represented by pastures located at higher elevations to which animals are taken to graze (called “Nalla”). Some wild food plants are collected there and later brought home (Figure 5).

After a detailed literature survey, we found some food uses of certain plant taxa, which were quite new and have not been mentioned, to the best of our knowledge, in any previous wild food ethnobotanical reports in Pakistan and surrounding Pamir areas. These plants included: *Artemisia annua*, *Berberis parkeriana*, *Hedysarum falconeri, Iris hookeriana*, *Lepidium didymium* and *Saussurea lappa.*

### 3.3. Cross Cultural Analyses

**Figure 6:**
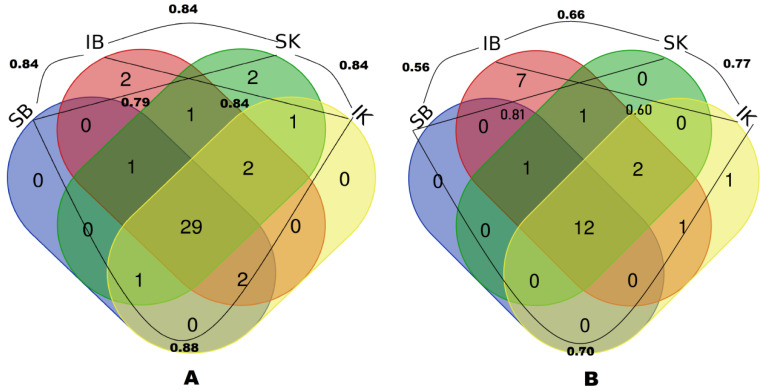
Venn diagrams showing the Jaccard indexes and overlap of (**A**) overall recorded wild food plants and (**B**) the most frequently reported wild food plants (quoted by more than 50% of the informants) among the four studied groups (IB: Ismaili Burusho, IK: Ismaili Kho, SB: Sunni Burusho, SK: Sunni Kho) in the Yasin Valley.

In the Yasin Valley, comparative assessment of wild plant uses among Ismaili Kho, Sunni Kho, Ismaili Burusho and Sunni Burusho indicated that the largest number of plant taxa was reported by Sunni Kho, and a large majority of wild food plant taxa were shared, apart from a few, minor divergences (Figure 6A). The greatest similarity was observed between Ismaili Kho and Sunni Burusho for recorded wild food plant uses.

In order to specify more precisely the effect of language and religion on traditional knowledge of wild plant uses, we also compared the most frequently quoted plants among the groups. It was noted that half of the frequently reported taxa were common to all the studied groups and the majority of these plants were reported by Ismaili Burusho (Figure 6B).

**Figure 7:**
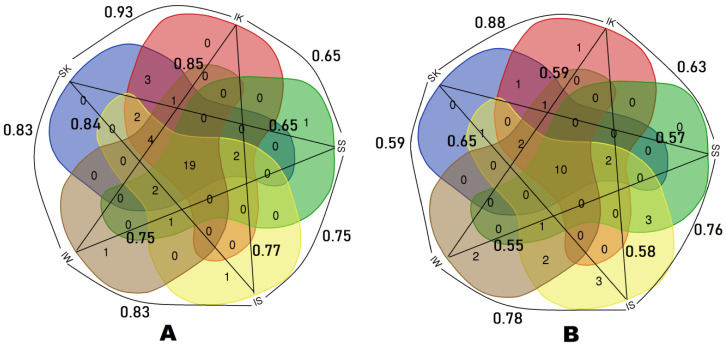
Venn diagrams showing Jaccard indexes and the overlap of (**A**) overall recorded wild food plants and (**B**) the most frequently reported wild food plants (quoted by more than 50% of the informants) among the five studied groups (IK: Ismaili Kho, IS: Ismaili Shina, IW: Ismaili Wakhi, SK: Sunni Kho, SS: Sunni Shina) in the Ishkoman Valley.

In the Ishkoman Valley, cross-cultural comparison of wild food plant uses among Ismaili Kho, Sunni Kho, Ismaili Shina, Sunni Shina and Ismaili Wakhi demonstrated that of the total reported taxa half were common among the groups (Figure 7A).

**Figure 8:**
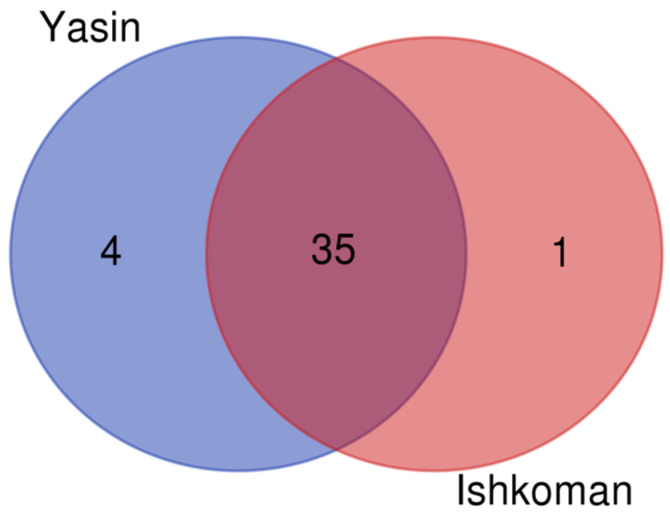
Venn diagram showing the overlap among the studied groups in both valleys for the recorded wild food plants.

The largest number of wild plant taxa was reported by Sunni Kho, while Sunni Shina reported the lowest number of wild food taxa. The greatest similarity was observed between Ismaili Kho and Sunni Kho, whereas the least similarity was recorded between (a) Sunni Kho and Sunni Shina, and (b) Ismaili Kho and Ismaili Wakhi. Furthermore, in the valley, approximately one third of the frequently quoted plant taxa were used by all the studied groups (Figure 7B). Similarly, we also found great overlap of wild food taxa reported from both valleys, and approximately 90% of the plant uses were the same (Figure 8).

## 4. Discussion

### 4.1. Ismailism and Its Cultural Pluralism: A Homogenizing Factor for LEK and LFK?

The cross-cultural ethnobotanical analysis has shown that remarkable commonalities among the selected studied groups and the overlapping pattern of wild plant uses confirms some form of cross-interaction among communities, which shared the same environmental and sociocultural space for a few centuries. Our main finding suggests that the heritages of LEK and LFK concerning WFPs of the considered groups have not remained distinct and this could be due to intense social exchanges, which possibly the dominant Ismailism in the area could have allowed across history [76]. Moreover, in other works we have suggested that intermarriages between different cultural groups may lead to the homogenization of traditional knowledge, since most LEK and LFK are shaped by transmission passing from one female generation to the next [23,24,25,30]. In order to better evaluate the impact of this variable on the transmission of this knowledge system, we also qualitatively addressed kinship relationships among individuals of the studied groups. While study participants confirmed that—as nearly everywhere in the world—the transmission of LEK and LFK systems regarding food plants (and especially wild vegetables) passes mainly from mothers to daughters, they did not openly recognize the presence of endogamic rules. We often noticed the presence of intermarriages among the different cultural groups; for example, during the field research in the Ismaili community we found that a Burusho man married a Kho woman and a Shina woman married a Kho man. However, it was not uncommon to observe Sunnis that intermarried with locals having perhaps a different language but sharing the same faith. We also found families in which some members belonged to the Ismaili faith of Islam while others converted to the Sunni faith. These observations suggest a possible trend among Sunnis, which contradicts the historical cultural pluralism forged over centuries by Ismailism.

However, study participants confirmed us in their narratives that knowledge on WFPs goes beyond linguistic and religious boundaries and they have the perception that they all follow the same food patterns. During the survey, participants did not report any WFPs, which were exclusively consumed during specific food events or religious festivities of individual communities.

It is worth mentioning that among all the studied groups, Burusho was the only autochthonous linguistic group in the area, which is considered an isolated language group; Burushaski speakers used some WPFs that were very frequently reported within their group and hardly used by the other groups. This confirms the peculiar position of this group within the study area, where historically diverse invaders pushed the Burusho into the most remote and highest areas of the valley. Burushaski speakers were originally isolated, and yet the fact that they share qualitatively (but not quantitatively and in their frequency) the same wild food plants gathered by the other groups shows that after the arrival of other groups into the area they possibly generated complex cultural adaptation and negotiation processes. The crucial role of these negotiations among diverse ethnic pastoralist groups in the mountains of NW Pakistan was superbly described by Fredrick Barth (1928–2016) half a century ago [77].

Kassam has analyzed how adaptation is fundamentally linked to indigenous cultural values and local wisdom, and despite the fact that local communities in the neighboring Pamir Mountains have been subjected to dramatic changes within the last century (colonization, the Cold War, penetration of the market system, civil war and, lastly, dramatic climate change), their survival and continued existence speaks to their amazing capacity to adapt, demonstrating cultural and ecological pluralism, and highlighting the importance of “keeping all the parts” [78,79].

On the other hand, in another study we pointed out that selective convergence in wild food ethnobotanical knowledge could occur between ethnic groups during periods of food insecurity, and may leave a lasting mark on the body of orally transmitted LEK and LFK [28]. This could have also happened in the study area considered here.

Moreover, LEK and LFK may have been influenced by a specific linguistic adaptation as well. For instance, it was observed that the local names of certain WPFs are common to all the different communities: *Capparis spinosa* is known as Kaveer by the Kho and Shina, *Carum carvi* is referred to as Hojooj by the Burusho, Kho and Shina, *Chenopodium album* is called Konakh by both the Burusho and Kho and *Eremurus himalaicus* was described as Laqa by the Burusho, Kho and Shina. This linguistic adaptation was possibly linked to a broader cultural adaptation that minority groups underwent toward the majority groups or the groups speaking the lingua franca (Kho in our study area, [49]).

### 4.2. Dynamics of Change of Local Knowledge: Nalla and Its Vanishing Role

LEK and LFK regarding WFPs has gone through remarkable changes in the past few decades, not only in terms of the frequency of gathering, but also generational dynamics in WFP gathering. For instance, participants frequently mentioned that in the past wild garlic (*Allium fedtschenkoanum* and *A. carolinianum*) was used in traditional cooking instead of onions bought from the market, but now no one uses this ingredient on a daily basis anymore. Similarly, *Urtica dioica* was formerly used as a common vegetable, but study participants claimed that the plant is no longer used very often in this manner. Participants also frequently claimed that the consumption of wild food plants has reduced with the passage of time due to certain factors including the availability of cultivated vegetables both from the market and fields. Some participants mentioned that most of the wild plants are not available near houses or within the village. In the study area, especially in the Yasin Valley, the Burusho community retains extensive knowledge of wild food plants. People living in Nalla, which still represent the core environment of the original pastoralist socioecological system, have broad skills in recognizing and gathering WFPs, but there is also a threat to this knowledge as they have begun to move down to the plain areas of the valleys in search of jobs and business opportunities. The majority of the visited local communities no longer pay attention to their wild food resources, apart from those community members still living in Nalla during the summer.

One of the study participants (71-year-old man) from the Yasin Valley mentioned his views using these words:

“My father and grandfather used wild food plants in the old days when we were children and we also used the mountain, taking our animals to graze over there. When I was young I could walk and I was able to go to the mountains but now I can’t climb them. As you ask about the degradation of wild food plants, I am not sure whether wild food plants are increasing or decreasing. I am old and my legs don’t allow me to go there and see over there. Our younger generation is not interested in such cultural things because they have facilities and can get a modern education in cities. But I think that wild plants are still available in Nalla, but who among us is going to Nalla? Of course no one goes there because we don’t need wild food plants anymore and the consumption of wild food plants is a story of former times when my father and forefather were poor and wild plants were easily available”.

During the survey we also observed that some locals gathered WPFs at different locations across the region (Figure 5) because they also recognize their medicinal value and thus their practical and economic importance. Some locals mentioned that previously these plants were collected for food purposes but now they were collected for mainly medicinal use only; this was the case, for example, for *Allium carolinianum, Capparis spinosa* and *Bergenia stracheyi*.

*Allium carolinianum* is generally preferred by elderly people for treating joint pain. *Capparis spinosa*, which is found near the houses within villages, is frequently gathered in order to obtain an extract from its flowers; this extract, which was found in many homes, is famous for treating liver problems, as well as diabetes, hepatitis, cough, cold, fever and malaria. Similarly, in some households we also found the dried rhizomes of *Bergenia stracheyi*. Participants used to prepare an herbal tea of these rhizomes, but now they mainly utilized them for gastric problems. Local inhabitants also go to *Nalla* to gather these wild food plants for medicinal purposes and if they find them easily then the plants are cooked and eaten as a meal as well. During the course of the study, we recorded an interesting local perception about a wild plant, which is well known in mountain areas of Central Asia as an important, but threatened medicinal plant: *Saussurea lappa*, which is locally known as “Minal”. Participants reported that in the past this plant was added to milk or yogurt in order to increase the amount of fat in yogurt or butter, respectively; this species was only been used within some specific families locally known as “Khandani Khalaq”. It was believed that if a household used Minal this would have automatically reduced the amount of butter in the nearby houses, which was morally unacceptable and not allowed from a religious point of view. This suggests how certain food taboos in the study area may have been associated to the use of rare natural resources such as *Saussurea*. Similar patterns were recently observed in the gathering of ritual plants in Benin [80] and were especially well described by Alpina Begossi in her pioneering work on the coevolutive significance of fishing rare species in Brazil [81].

Moreover, in the study area locals mentioned that now Minal is used only for medicinal purposes in the Ghizar region and in other areas of Gilgit-Baltistan, as also well described in recent ethnobotanical literature [67].

This shift from of WFPs into the medicinal domain may have different reasons: the gathering of wild medicinal represents a good source of cash, while the resilience of folk medicinal practices seems to find wider social acceptance than that of WFPs, which often represent symbols of economic marginalization.

### 4.3. Strategies for the Future: Revitalizing the Wild Food Cultural Heritage and Nalla

In the study area, transformation of subsistence economies has led to market dependency, which has greatly affected local food systems. Due to this change, local knowledge systems are facing critical problems, which in turn are posing biocultural conservation problems and need critical attention from stakeholders. People living in Nalla still retain an important portion of LEK, but this heritage is threatened, since the Nalla-centered socioecological system is vanishing as a result of the increasing migration to the plain areas of the valleys for employment. In order to dynamically preserve the biocultural heritage and foodscape and to empower local subsistence economies, it is urgent that measures be put in place in order to revitalize Nalla. Policy makers and various stakeholders should also pay attention to the issue of how local knowledge systems, especially those circulating around Nalla, could be promoted and further transmitted. For fostering the resilience of LEK, it may be necessary to incorporate it into the existing school curricula with the help of traditional knowledge holders. This would not only possibly further transform LEK, but would also increase awareness of the need to preserve the local natural resources and especially the complex socioecological systems attached to it.

Revitalizing and increasing the appreciation for local wild plant resources could have a positive impact on the promotion of sustainable rural development. The entire area could become a hotspot of ecotourism, improving the economic condition of the local people by honoring their local culture. Ecotourism in the region could also alleviate the social isolation of some local groups and provide additional awareness of their traditional food cultural heritage, which could help to prevent the further erosion of LEK. However, promoting wild species could also have a negative impact on available genetic resources (over-exploitation) and therefore it would be crucial to frame this strategy within the larger picture of environmental education. Designing community-centered biocultural diversity conservation projects based on LEK could possibly generate better outcomes than traditional biodiversity conservation strategies. Nalla could become the center of these bio-cultural initiatives, in which local communities could engage with urban civil societies and visitors to also foster virtuous social exchange and internal social cohesion. It would be equally interesting to examine how younger generations of the area re-articulate this knowledge related to wild plant taxa as younger individuals are more exposed to “modernization” and are less interested in maintaining this biocultural heritage.

### 4.4. Food Security in Rural Areas of Pakistan: Quo Vadis?

In mountainous areas of Pakistan, as in other developing areas, livelihoods were based mainly on subsistence horticulture, livestock rearing and the use of common pastures, rangeland and forests. However, in recent times, governmental wheat subsidies and facilitated access to city markets have led to a profound transformation of livelihoods and farming systems, as in other communities of the region [34]. Until the 1980s, farming was mainly subsistence-oriented and modern technologies were largely absent, with dominant crops being wheat, barley, millets, buckwheat, maize, alfalfa and apricots. Over the last four decades, new technologies have been adopted, traditional crops have been replaced by potatoes and other cash crops, and small-scale family agriculture has lost its prominent importance in the diversified livelihoods of the village population [82]. Obviously these changes have also improved access to food supplies beyond local production. However, the aforementioned socioeconomic changes have led to a loss of food sovereignty as well, i.e., the self-sufficiency of local communities has decreased, posing new risks related to market dependency and also environmental hazards [83,84]. For instance, in Gilgit-Baltistan, a recent historic event has been critical for subsequent changes in local food systems: the catastrophic Attabad landslide in 2010 cut off a large populated area from access to down country Pakistan and posed serious economic threats for local communities across the area. This raises the question of whether the developments since the 1970s have actually reduced the ability of local food systems to cope with emerging economic, political and environmental risks and challenges [85]. Ultimately, food security issues in mountainous and rural areas of Pakistan still largely need to be addressed from a broader perspective, since food vulnerability is not only a matter concerning agroecosystems, supply chains, and even the resilience of LEK and LFK regarding WFPs. In particular, food insecurity in Pakistan also involves a complex interplay in which diverse factors are determinant: environmental change and degradation, cultural changes, gender issues (i.e., the still largely invisible social and economic role of women [86]), as well as the overall governance of food policies and economies at both the regional and national level [87,88].

## 5. Conclusions

The field study conducted among the different linguistic and religious groups living in the Ishkoman and Yasin valleys revealed a considerable, but possibly eroded, LEK and LFK concerning the folk use of wild botanical and mycological taxa. The finding showed that the studied groups of both valleys are currently mainly attached to horticultural food ingredients and dairy coming from livestock rearing, yet only partially still rely on wild food plant taxa. More than half of the wild food plant reports referred to vegetables that are cooked, while snacks roughly represented one third of the recorded botanicals. Comparative analysis of wild plant uses among the different groups in the two valleys showed no significant variation among the diverse visited groups. The conducted literature review demonstrated that some wild food plant taxa recorded in the current field study were not reported in any previous ethnobotanical food studies from the region. It is important to note that in this study there were some plant taxa recorded with past uses, while others switched from being food species to medicinal species. This study addressed also the complex transformations LEK and LFK systems underwent during the past decades and the possible strategies that are needed for revitalizing the complex wild food biocultural heritage, as well as for better implementing the future food security in mountain areas of Pakistan.

Further cross-cultural and possibly cross-temporal food ethnographic studies among different groups living in other areas of the Hindukush range could be vital for contributing to a better understanding of the dynamics of change in ethnobotanical knowledge and foodways in disadvantaged mountain areas.

## Figures and Tables

**Table 1:** Characteristics of the visited mountain villages and studied communities.

Language	Village	Elevation (Metres Above Sea Level)	Approx. Number of Inhabitants	Number of Interviewees (Male/Female)	Islamic Faith	Arrival in the Area	Subsistence Activities
Yasin Valley							
Burushaski	Barkolti	2462	12,000	5/5	Sunni	Autochthonous	Horticulturalism and pastoralism
				-	Ismaili		
	Ghojalti	2415	9000	-	Sunni		
				5	Ismaili		
	Sandi	2395	7000	-	Sunni		
				3/2	Ismaili		
	Sultan Abad	2405	3000	-	Sunni		
				3/6	Ismaili		
	Thawoos	2397	4000	3/2	Sunni		
				1/1	Ismaili		
	Yasin Khaas	2371	7000	3/2	Sunni		
				-	Ismaili		
Khowar	Barkolti	2462	12,000	-	Sunni	Arrived in the 17th century from Chitral, North-West Pakistan	Horticulturalism and pastoralism
				-	Ismaili		
	Ghojalti	2415	9000	-	Sunni		
				-	Ismaili		
	Sandi	2395	7000	2/1	Sunni		
				2/2	Ismaili		
	Sultan Abad	2405	3000	1/2	Sunni		
				5/3	Ismaili		
	Thawoos	2397	4000	2/2	Sunni		
				4/4	Ismaili		
	Yasin Khaas	2371	7000	7/3	Sunni		
				-	Ismaili		
Ishkoman Valley							
Khowar	Chatorkhand	2092	6000	13/7	Sunni	Arrived in the 17th century from Chitral, North-West Pakistan	Horticulturalism and pastoralism
				10/10	Ismaili		
Shina	Ishkoman Khaas	2092	3000	15/5	Sunni	Arrived from other areas of Gilgit-Baltistan (North Pakistan) in the late 18th century	Mainly horticulturalism
				12/8	Ismaili		
Wakhi	Imit	2391	3500	-	Sunni	Migrated into the area from Wakhan Corridor (North-East Afghanistan) during the 19th century	Pastoralism and horticulturalism
				12/8	Ismaili		

**Table 2:** Gathered wild food plants recorded in the study area.

Botanical Taxon; Family; Botanical Voucher Specimen Code	Recorded Local Names	Parts Used	Recorded Local Food Uses	Religious and Linguistic Communities in Which the Food Use Was Recorded									Previously Reported in Pakistan
				IBY	IKI	IKY	ISI	IWI	SBY	SKI	SKY	SSI	
*Allium fedtschenkoanum* Regel; Amaryllidaceae; SWAT005487	Gasho ^B^ Kasch ^K^ Karghapyaz ^W^ Khasho ^S^ Teshto ^K^	Aerial parts	Cooked	+	+	+	+	+	+	+	+	+	Yes
*Allium carolinianum* DC.; Amaryllidaceae	Latruk ^K^ Lanturk ^W^ Khasch ^B,S^	Aerial parts	Cooked	+	+	+	+	+	+	+	+	+	Yes
*Amaranthus cruentus* L.; Amaranthaceae; SWAT005512	Bardoomhoi ^B^ Kruishakh ^K, S^ Lolosha ^S^ Sakarghaz ^W^	Leaves	Cooked	+	+	+	+	+	+	+	+	+	Yes
*Artemisia annua* L.; Asteraceae; SWAT005779	Kakasho ^B, S^ Khalkhalich ^K^ Khulkhulo ^K^ Stwirg ^W^	Aerial parts	Cooked in *Dawdoo*	+	+	+	+	+	+	+			No
*Berberis parkeriana* C.K. Schneid.; Berberidaceae; SWAT005491	Chong ^K^ Chikoring ^S^ Ishkor ^W^ Ishkoring ^B^ Ishkorash ^B^ Ishkoring ^K^ Karaqoot ^W^ Zolag^W^	Fruits	Raw snack	+	+	+	+	+	+	+	+		No
*Bergenia stracheyi* (Hook. F. and Thomson) Engl.; Saxifragaceae	Beesapur ^K^ Bushk ^W^ Geesapur ^K^ Geesapur ^B^ Geesapur ^S^ Sapur ^W^	Roots	Tea	+	+	+	+	+	+	+	+	+	Yes
*Brassica rapa* L.; Brassicaceae; SWAT005807, SWAT005520	Charrsham ^S^ Chiroogh ^W^ Malharo ^B^ Malharo ^K^	Leaves	Cooked	+	+	+	+	+	+	+	+		Yes
*Capparis spinosa* L.; Capparaceae; SWAT005794	Chopur ^B^ Kapur ^W^ Kaveer ^K^ Kaveer ^S^	Flowers	Cooked	+	+	+	+	+	+	+	+	+	Yes
*Carum carvi* L.; Apiaceae; SWAT005486	Hojooj ^B^ Hojooj ^K^ Hojooj ^S^ Zeera ^W^	Seeds	Seasoning	+	+	+	+	+	+	+	+	+	Yes
			Tea	+	+	+	+	+	+	+	+	+	
*Chenopodium album* L.; Amaranthaceae; SWAT005509, SWAT005499	Konah ^S^ Konakh ^K^ Konakh ^B^ Shileet ^W^	Aerial parts	Cooked	+	+	+	+	+	+	+	+	+	Yes
*Cotoneaster nummularius* Fish. and Mey.; Rosaceae; SWAT005485	Dodool ^S^ Dundal ^B^ Mikeen ^K^	Fruits	Snack	+	+	+	+	+	+	+	+	+	Yes
*Echinops echinatus* Roxb.; Asteraceae; SWAT005490	Chacheer ^S^ Chancheer ^B^ Chancheerak ^K^ Kankeer ^W^ Kareer ^W^	Roots	Snack	+	+	+	+	+	+	+	+	+	No
*Elaeagnus angustifolia* L.; Elaeagnaceae; SWAT005806, SWAT005808	Ginahoor ^B^ Gunair ^S^ Sisk ^W^ Sonjoor ^K^	Bark	Tea	+	+	+	+	+	+	+	+	+	Yes
		Flowers	Seasoning	+	+	+			+	+	+		
		Fruits	Snack	+	+	+	+	+	+	+	+	+	
*Eremurus himalaicus* Baker; Xanthorrhoeaceae	Laqa ^K, S^ Laqa ^B^ Laqanz ^K^ Laqo ^S^	Aerial parts	Cooked	+	+	+	+	+	+	+	+	+	Yes
*Hedysarum falconeri* Baker; Leguminosae	Shavoo ^K^ Shingalo ^B^	Bark	Tea	+		+			+		+		Yes
		Shoots	Snack	+		+			+		+		
*Helianthus tuberosus* L.; Asteraceae; SWAT005476	Jangli Kachalo ^K, S^ Jangli Kachalo ^B^	Tubers	Snack	+	+	+	+	+	+	+	+		No
*Iris hookeriana* Foster; Iridaceae; SWAT005478	Shato ^K^ Sosan ^B, K^ Sosan ^W, S^	Aerial parts	Cooked	+		+	+	+	+	+	+	+	No
*Lepidium didymium* L.; Brassicaceae	Holominazk i^B^ Khodong ^K^ Shadoi ^W^ Shadong ^K^ Shadong ^K^ Shadoging ^S^	Aerial parts	Cooked	+		+	+	+	+	+	+	+	No
*Lepyrodiclis holosteoides* (C.A. Mey.) Fenzl ex Fisch. and C.A. Mey.; Caryophyllaceae	Balghar ^B^ Birghal ^K, S^ Yarkwoosh ^W^	Aerial parts	Cooked	+	+	+	+	+	+	+	+	+	Yes
*Medicago sativa* L.; Fabaceae; SWAT005797, SWAT005795	Ishpit ^B^	Aerial parts	Cooked	+									Yes
*Mentha longifolia* (L.) L.; Lamiaceae; SWAT005792, SWAT005790	Ben ^K^ Phalaling ^B^ Phaleel ^S^ Wadan ^W^	Aerial parts	Salad	+	+	+	+	+	+	+	+	+	Yes
*Morchella esculenta* (L.) Pers.; Morchellaceae	Shalkhot ^W^ Shoto ^B, K^ Shoot ^S^	Aerial parts	Cooked	+	+	+	+	+	+	+	+	+	Yes
*Portulaca quadrifida* L.; Portulacaceae	Pichili ^W, S^ Pichiling ^K, B^	Aerial parts	Cooked	+	+	+	+	+	+	+	+	+	Yes
*Rheum maximowiczii* Royle; Polygonaceae	Chotal ^S^ Naik ^W^ Shpaar ^K^ Zeekap ^B, K^ Zeekap ^K, S^ Zeepak ^S^	Stalks	Snack	+	+	+	+	+	+	+	+	+	Yes
*Rheum* sp.; Polygonaceae	Chotool ^S^ Kakool ^K, B^ Naik ^W^,	Young shoots	Snack	+	+	+	+	+	+	+	+	+	Yes
*Ribes alpestre* Wall.ex. Decne.; Grossulariaceae; SWAT005775	Chilazum ^W^ Ishkorash ^B^ Shatoo ^K^ Shatoo ^B^ Shatoo ^K, S^	Fruits	Snack	+			+	+	+		+	+	Yes
*Ribes* sp.; Grossulariaceae; SWAT005774	Ginat ^W^ Inaat ^W^	Fruits	Snack					+					Yes
*Rosa webbiana* Wall. ex Royle; Rosaceae; SWAT005502	Chareer ^W^ Goshalgoo ^S^ Shawo ^B^ Shinai ^S^, Shingai ^S^ Throni ^K^	Bark	Tea	+	+	+	+	+	+	+	+	+	Yes
		Flowers	Tea	+	+	+	+	+	+	+	+	+	
			Seasoning	+	+	+	+	+	+	+	+	+	
*Rubus fruticosus* G.N. Jones; Rosaceae	Tikbaranj ^B^ Chootimirach ^K^ Marach ^K^ Marooch ^K^	Fruits	Snack	+	+					+	+		Yes
*Rumex dentatus* L.; Polygonaceae; SWAT005468	Sirkonzoor ^K^	Leaves	Cooked		+	+				+	+		Yes
*Saussurea lappa* (Decne.) C.B. Clarke; Asteraceae	Minal ^B, K, S^	Aerial parts	Additive in the home-made processes of yogurt and butter production	+	+	+			+	+	+		No
*Salvia nubicola* Wall. ex Sweet; Lamiaceae; SWAT005761	Paltasho ^B^	Shoots	Snack	+									Yes
*Silene conoidea* L.; Caryophyllaceae; SWAT005481, SWAT005514	Hapupar ^B, K, S^	Aerial parts	Cooked	+	+	+	+		+	+	+		Yes
*Silene vulgaris* (Moench) Garcke; Caryophyllaceae; SWAT005475	Tumtak ^K, S^	Aerial parts	Cooked		+		+			+	+	+	Yes
*Sorbus* sp.; Rosaceae	Tar bhalt ^B^ Lalmi Palough ^K^	Fruits	Snack	+							+		Yes
*Taraxacum campylodes* G.E. Haglund; Asteraceae	Ishkanacho ^B, K, S^ Papas ^W^	Leaves	Cooked and salad	+	+	+	+	+	+	+	+	+	Yes
*Thymus linearis* Benth.; Lamiaceae	Jambilak ^W^ Krotum ^S^ Sew ^K^ Tumoor ^B, K^ Tumooru ^S^	Aerial parts	Tea and seasoning	+	+	+	+	+	+	+	+	+	Yes
*Tulipa* sp.; Liliaceae	Cheeram ^K^ Chirongo ^B^	Bulbs	Snack	+		+			+		+		Yes
*Urtica dioica* L.; Urticaceae; SWAT005479, SWAT005501	Drozono ^K^ Ghashoshing ^B, K^ Joomi ^S^ Zoomi ^S^	Leaves	Cooked	+	+	+	+		+	+	+	+	Yes
*Vicia sativa* L.; Fabaceae; SWAT005798, SWAT005799	Barawo ^B, K, S^	Aerial parts	Cooked	+	+	+	+			+	+		Yes
Unidentified taxon	Navoohar ^B, K, S^	Aerial parts	Cooked	+		+	+		+		+		

^B^: Folk name recorded among Burusho people; ^K^: folk name recorded among Kho people; ^S^: folk name recorded among Shina people; ^W^: folk name recorded among Wakhi people; IBY: food use recorded among Ismaili Burusho in the Yasin Valley; IKI: food use recorded among Ismaili Kho in the Ishkoman Valley; IKY: food use recorded among Ismaili Kho in the Yasin Valley; ISI: food use recorded among Ismaili Shina in the Ishkoman Valley; IWI: food use recorded among Ismaili Wakhis in the Ishkoman Valley; SBY: food use recorded among Sunni Burusho in the Yasin Valley; SKI: food use recorded among Sunni Kho in the Ishkoman Valley; SKY: food use recorded among Sunni Kho in the Yasin Valley; SSI: food use recorded among Sunni Shina in the Ishkoman Valley; +: food use quoted by less than 50% of the study participants; **+**: food use quoted by 50% or more of the study participants; *Dawdoo:* noodle soup traditionally consumed in the winter season.
